# P-2340. Single-Center Inpatient Measles Analysis During a Congregate Setting Outbreak; Comparing Vaccine History between Measles Positive and Negative Cohorts

**DOI:** 10.1093/ofid/ofae631.2492

**Published:** 2025-01-29

**Authors:** Marielle J Fricchione, David Nguyen, Colleen B Nash

**Affiliations:** Rush University Medical Center, Chicago, Illinois; Rush University Medical Center, Chicago, Illinois; Rush University Medical Center, Chicago, Illinois

## Abstract

**Background:**

Chicago’s 2024 measles outbreak affected many new arrivals living in congregate settings with unknown or poorly documented vaccination history. Despite post-exposure prophylaxis provided at shelters, many families still developed measles. Frequency of measles exposure in congregate settings certainly plays a role in infection, but vaccination history and source should also be evaluated.

**Table.**
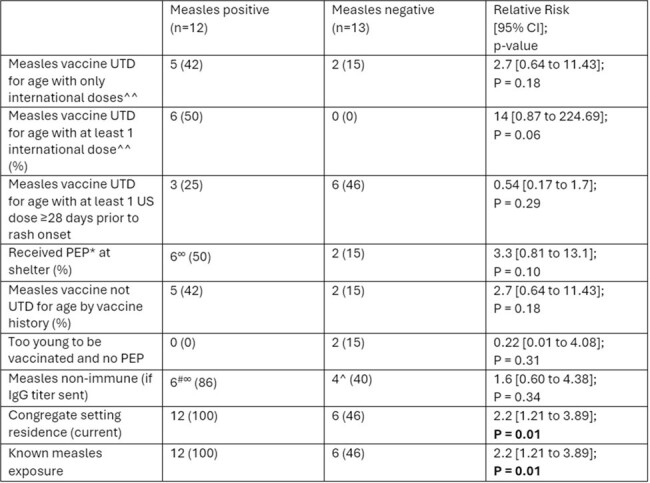
Characteristics of Measles Patients Under Investigation (PUI) stratified by PCR result

* One case received IGIM and five received 1 MMR post-exposure prophylaxis dose given at the shelter between 10—30 days prior to rash onset (median: 11 days), ∞ including vaccine strain case, # n=7, ^n=10, ^^Countries of origin: Venezuela, Peru, Ecuador

**Methods:**

Data was abstracted from 12 measles PCR positive and 13 measles PCR negative patient charts from 3/10/24—4/23/24 including measles vaccination history, IgG antibody titers, concurrent infections and measles complications. Relative risks and p-values were calculated using MedCalc Software Ltd (Version 22.023).

**Results:**

From 3/10/24—4/23/24, 12 measles PCR positive patients were admitted with median age 16 years (range 13 months—43 years) including 3 parent-child dyads; serotyping through the Illinois Department of Public Health reported one vaccine strain and 11 wild type cases. Of the 11 wild type measles cases, 3 (27%) had concurrent infections; (2 of 3 group A streptococcus culture positive (oropharynx); 1 of 3 was rhinovirus/enterovirus positive (nasopharyngeal PCR); 8 of 11 (73%) had at least 1 measles complication including dehydration (4), acute otitis media (3), pneumonia (1), and hepatitis (2). Comparing vaccine history between measles negative and positive cohorts, there was a non-significant trend towards history of international vaccination in the measles positive cohort. History of current congregate setting residence and exposure to a known measles case were significantly associated with measles positivity with a relative risk of 2.2.

**Conclusion:**

Inpatient data is lacking during the 2024 international measles surge and new arrivals' vaccination history documentation is inconsistent. A trend towards international measles vaccine receipt in a measles positive cohort highlights the importance of US public health efforts to offer MMR vaccination to new arrivals. Limitations of this report include small cohort sizes and recall bias. Additional data is needed to describe the impact of improper vaccine storage and handling on vaccine effectiveness in the current international measles epidemic.

**Disclosures:**

All Authors: No reported disclosures

